# Recent Management Option for Hemorrhagic Stroke during Direct Oral Anticoagulant Therapy

**DOI:** 10.31662/jmaj.2023-0119

**Published:** 2023-09-25

**Authors:** Miki Fujimura

**Affiliations:** 1 Department of Neurosurgery, Hokkaido University Graduate School of Medicine, Sapporo, Japan

**Keywords:** hemorrhagic stroke, hemorrhagic infarction, anticoagulant therapy

The management of massive intracerebral hemorrhage and/or hemorrhagic infarction during antithrombotic therapy is the most challenging condition in neurosurgical practice, which requires urgent decompression and secure hemostasis. According to the marked increase in the elderly population in Japan, an increasing number of patients were administered with anticoagulant agents, either for secondary prevention of ischemic stroke or primary prevention in patients with nonvalvular arterial fibrillation. Patients with hemorrhagic stroke or hemorrhagic conversion of ischemic stroke during anticoagulant therapy were reported to have poorer clinical outcomes owing to the difficulty in acute neurosurgical management, but the risk assessment for hemorrhagic events associated with anticoagulants in the central nervous system is not easy ^(1)^.

In this difficult clinical condition, the authors successfully managed a patient with massive hemorrhagic infarction during direct oral anticoagulant medication of apixaban by two-stage surgery using intracranial pressure monitoring ^(2)^. The authors promptly performed the partial removal of hematoma with simultaneous administration of fresh frozen plasma, leading to an emergent reduction of intracranial pressure and then safely attempted radical decompressive craniectomy after the effect of apixaban had diminished. The authors’ two-stage management strategy was further justified by the continuous monitoring of intracranial pressure between first- and second-stage surgeries, and the favorable staged decompression was demonstrated by serial computed tomographic scans ^(2)^. While considering the recent advances in the neuroendoscopic modalities, the application of neuroendoscopic partial removal of the hematoma could have been a management option in the first-stage surgery in this case. It is necessary to evaluate the efficacy and safety of neuroendoscopic removal of intracranial hematoma in patients with anticoagulant administration in future studies.

Recently, Andexanet alfa, a factor Xa inhibitor that neutralizes the anticoagulant effect of factor Xa inhibitors, including apixaban, became available in Japan. Although Andexanet alfa was not available at the time in this reported case, this neutralizing agent is expected to significantly contribute to the management of the patients with hemorrhagic stroke, including hemorrhagic infarction managed by factor Xa inhibitors, such as apixaban and edoxaban ^(3)^. Based on the cumulative evidence, the most recent issue of the Japanese Guidelines for the Management of Stroke in 2023 recommends the administration of Andexanet alfa for the patients with hemorrhagic stroke using factor Xa inhibitors (Recommendation grade B) ^(4)^. Taken together, radical decompressive craniectomy with hematoma removal after the neutralization of factor Xa activity could be a management option if Andexanet alfa is available. Further investigation of a larger number of patients from multiple institutes is warranted to address this important issue.

## Article Information

### Conflicts of Interest

None

### Disclaimer

Miki Fujimura is one of the Editors of JMA Journal and on the journal’s Editorial Staff. He was not involved in the editorial evaluation or decision to accept this article for publication at all.
